# Survival of Human Bone Marrow Plasma Cells In Vitro Depends on the Support of the Stromal Cells, PI3K, and Canonical NF‐kappaB Signaling

**DOI:** 10.1002/eji.202451358

**Published:** 2025-01-08

**Authors:** Zehra Uyar‐Aydin, Shirin Kadler, Roland Lauster, Sina Bartfeld, Mark Rosowski

**Affiliations:** ^1^ Department Medical Biotechnology Institute of Biotechnology Technische Universität Berlin Berlin Germany; ^2^ Si‐M/Der Simulierte Mensch Technische Universität Berlin and Charité Universitätsmedizin Berlin Berlin Germany

**Keywords:** bone marrow, human culture model, memory cells, plasma cell

## Abstract

Contrary to short‐lived plasma cells, which survive only 3–5 days, long‐lived plasma cells (LLPCs) contribute to the humoral memory of the body and thus also to many antibody‐related diseases. The ability of plasma cells to persist over months, years, and even a lifetime has been demonstrated in vivo. Yet, the in vitro culture of human primary bone marrow‐derived plasma cells has been limited to a few days. Here, we establish culture conditions for human primary bone marrow‐derived plasma cells for 21 days. Plasma cells and stromal cells are isolated from human bone marrow and cultured in 2D or a 3D ceramic scaffold. The plasma cells’ survival and antibody secretion depend on direct contact with stromal cells. The culture promotes CD19‐negative PCs. Inhibition of the PI3K or NF‐kappaB pathways using chemical inhibitors reduced the survival of the plasma cells. These results underline the supportive role of the stromal cells for the survival of the LLPC and confirm mechanisms that were identified in mouse LLPCs also for human LLPCs. The culture described here will promote further studies to deepen our understanding of the human LLPC.

## Graphical abstract

This study established a 21‐day in vitro culture system for human long‐lived plasma cells (LLPCs), demonstrating their survival and antibody secretion depend on stromal cell contact. PI3K and NF‐kappaB pathways were crucial for LLPC survival, aligning with findings in mice. This model advances the understanding of human LLPC biology.

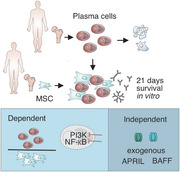

## 1 Introduction

After encounter of their stimulus, B‐lymphocytes will differentiate to become one of two types of antibody‐producing cells: either plasmablasts, which will undergo cell death after 3–5 days of antibody‐production, or long‐lived plasma cells (LLPCs), which persist for months, years or even lifelong. Continuous antibody secretion by LLPCs contributes to the humoral memory of the body [1, 2]. Alongside other long‐lived (memory) lymphocytes, LLPCs primarily reside in the human bone marrow (BM) [3, 4]. The survival of LLPCs depends on factors present in the bone marrow microenvironment since they show a short survival time upon extraction from their in vivo microenvironment [5, 6]. Low frequency (0.25%) among BM cells [7] limits the study of primary human BM‐derived LLPCs.

Factors of the niche for the survival of PCs in the BM were primarily defined based on mouse models. BM PCs reside in specialized niches comprising various cell types, extracellular matrix components, and secreted factors promoting their homing and survival [8, 9, 10].

Interacting cell types in vivo are mainly the BM reticular mesenchymal stromal cells (MSCs) [11, 12]. BM MSCs have been shown to support PC survival in in vitro co‐cultures [6]. Cell–cell contact is mediated by integrin/ligand interactions, which promote the survival of PCs, likely directly by the focal adhesion kinase and phosphatidylinositol 3‐kinase (PI3K) [13, 14, 15, 16].

Soluble factors of the bone marrow niche are either secreted by the stromal cell compartment or from accessory cells of the myeloid lineage [17]. Several factors, including interleukin (IL)‐6, C‐X‐C motif chemokine ligand (CXCL)12, a proliferation‐inducing ligand (APRIL) and B‐cell‐activating factor (BAFF), the latter both ligands for the B‐cell maturation antigen (BCMA) receptor, have been shown to promote plasma cell survival in mouse [5, 18, 19], as well as the generation of memory plasma cells in the bone marrow [20]. BCMA receptor signaling upregulates the antiapoptotic protein myeloid cell leukemia 1 (MCL‐1), which is essential in BM PC survival [21].

Existing in vitro human models enabling long‐term PC cultures are based on in vitro‐generated PCs that are differentiated from peripheral blood‐derived B cells, memory B cells, or antibody‐secreting cells after booster vaccination. Several groups have developed models for the blood‐derived or in vitro differentiation of plasma cells [22, 23, 24]. The maturation into LLPCs and their maintenance in these models required soluble factors secreted by the stromal cells of different origins (tonsil, BM) or of a mouse stromal cell line, in addition to different cytokines, for example, IL‐6, APRIL, or BAFF, interferon‐a, and IL‐21. The blood‐derived in vitro‐generated LLPCs are reprogrammed to a secretory phenotype and share many similarities with BM‐derived LLPCs, however, they are not identical to primary BM‐derived LLPCs [22]. Currently, there is a lack of human in vitro models mimicking the physiological niche microenvironment promoting the long‐term maintenance of human primary BM‐derived LLPCs.

In this study, we describe the in vitro maintenance of freshly isolated human BM LLPCs in co‐culture with primary human BM MSCs for 21 days. Transcriptional analysis of the MSCs indicates the expression of survival factors critical for PC maintenance. We further report the incorporation of the cells into a versatile and advanced 3D in vitro model based on a ceramic scaffold, allowing the integration of the model into a microfluidic device. This lays the groundwork for more complex and versatile applications in the future.

## 2 Results

### 2.1 Primary MSCs and PCs Isolated From Human Femoral Head Retain Characteristics In Vitro

**FIGURE 1:**
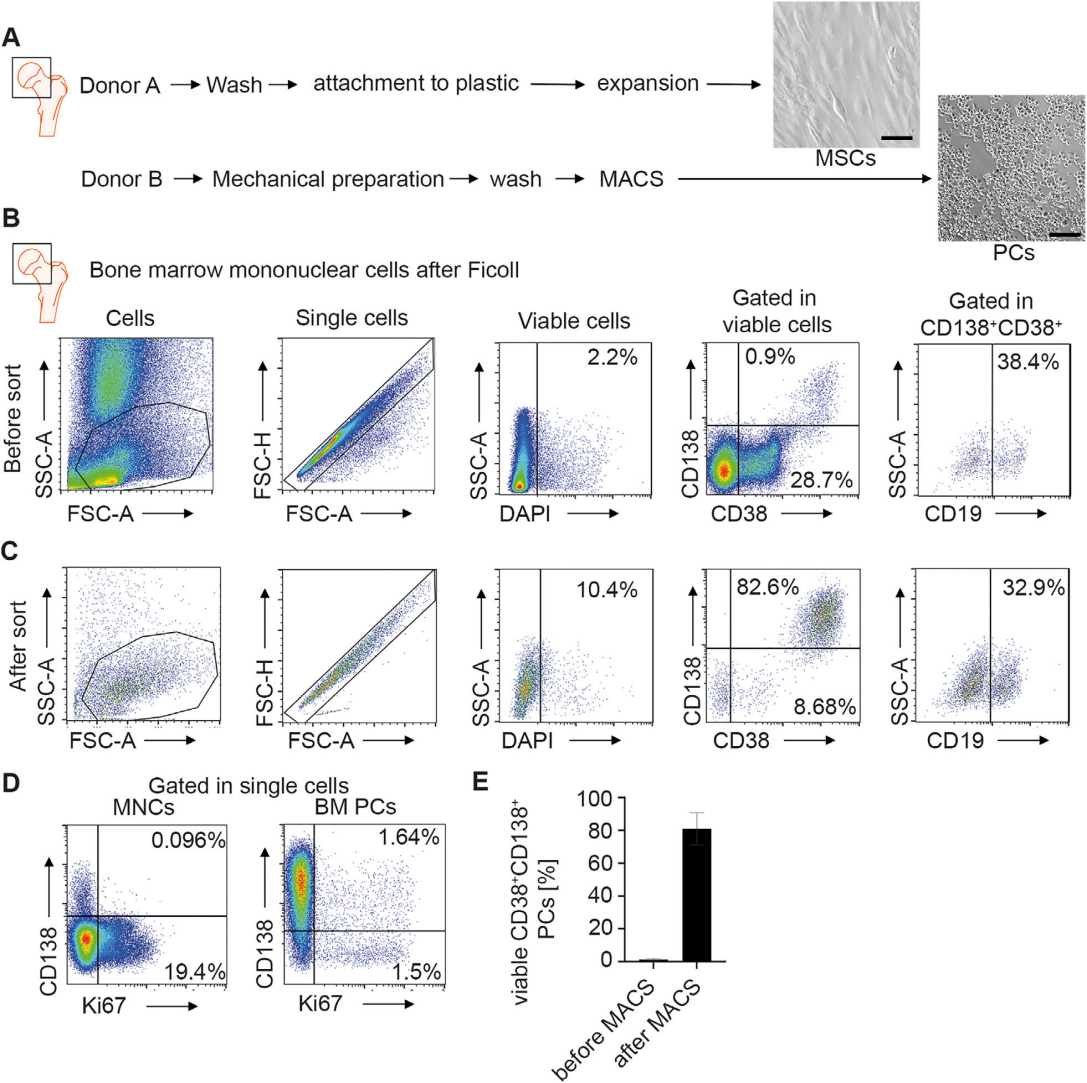
Isolation and characterization of PCs from the bone marrow of human femoral heads. (A) Scheme of isolation strategy. Human femoral heads and spongiosa were obtained after hip‐joint replacement surgery and mechanically disrupted. MSCs were selected by adherence to plastic. PCs were purified by a two‐step magnetic sorting. Brightfield microscopic image of MSCs after expansion and isolated plasma cells on day 1. Scale bars: 100 µm. (B, C) Representative flow cytometry analysis of viable CD38^+^CD138^+^ PCs and CD19 expression before (B) and after (C) the two‐step magnetic sorting. (D) Flow cytometric analysis of Ki67 expression shows a quiescent phenotype of isolated plasma cells. (E) Percentage of viable CD38^+^CD138^+^ PCs before the two‐step magnetic sorting (MACS) and afterward. Data in (B, C) are representative of at least 4 experiments. Data in (E) represent mean with SD of *n* = 25 donors.

To generate an in vitro culture model for human LLPCs, we isolated primary human PCs and MSCs from the bone marrow of human femoral heads obtained after hip joint replacement surgeries (Figure 1A). MSCs were isolated by density centrifugation and adherence to plastic and showed the typical spindle shape. Cultures were used after reaching confluency between passages 3 and 6. PCs were isolated by mechanical preparation of the femoral head and density centrifugation of the flushed‐out cell suspension (Figure 1A). Cells were magnetically sorted by depletion of non‐PCs, and the subsequent enrichment of cluster of differentiation (CD)138^+^ PCs (Figure 1B,C). PCs expressed the surface markers CD138 and CD38 and were Ki67‐negative, indicating their quiescence in terms of proliferation (Figure 1C,D). PCs showed heterogeneous expression of CD19 (Figure 1C). Purities of PCs reached about 80% (Figure 1E).

### 2.2 Human BM‐Derived Plasma Cells Are Maintained for 21 Days In Vitro

**FIGURE 2:**
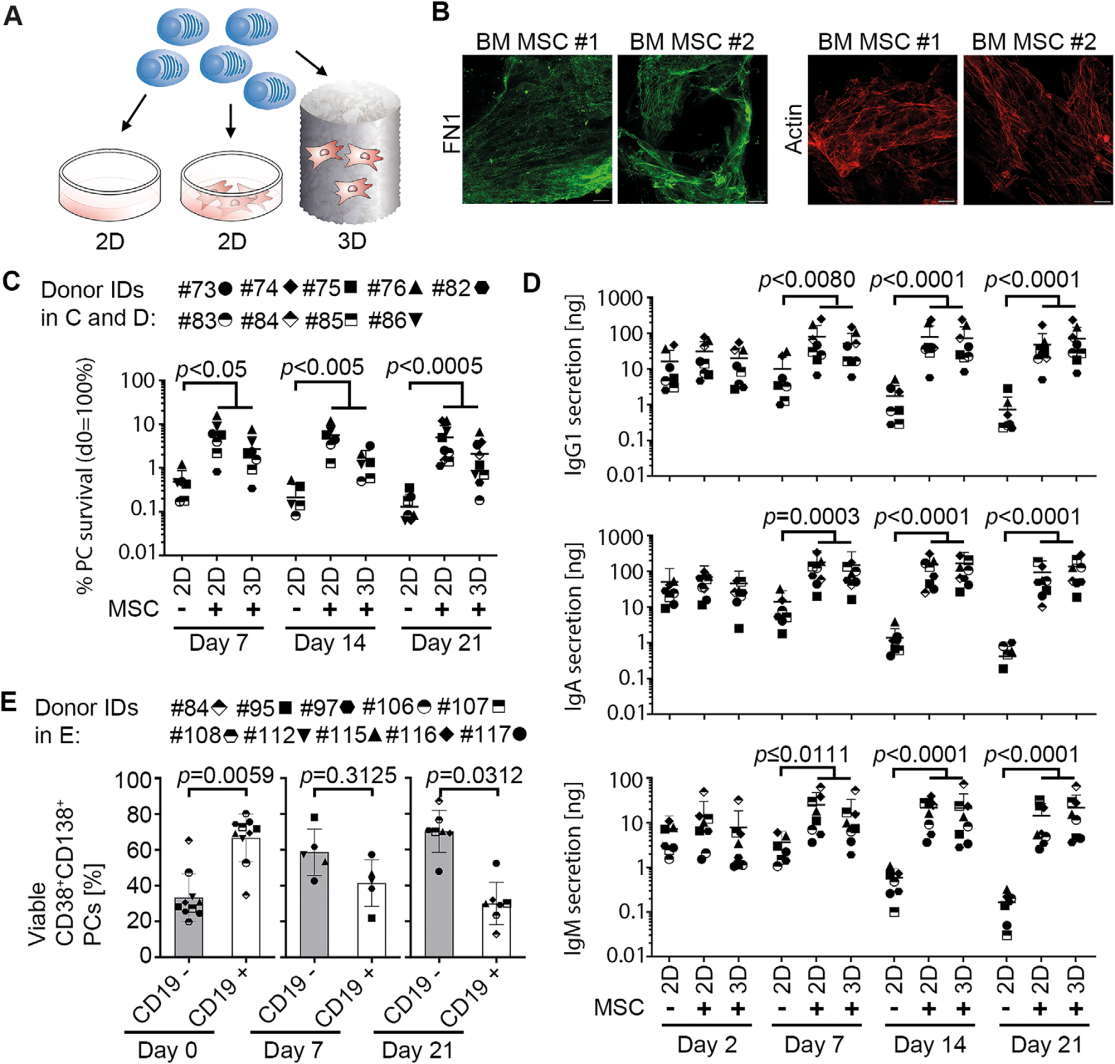
Functional human BM plasma cells are maintained for 21 days in vitro. (A) Schematic setup. Freshly isolated PCs were cultured for the indicated times without MSCs, on a 2D monolayer of primary MSCs (2D), or with MSCs pre‐seeded in the 3D BM ceramic scaffold (3D). (B) Confocal microscopic image of the 3D ceramic scaffold pre‐seeded with MSCs and subsequently co‐cultured with PCs for 7 days stained for fibronectin and actin (*n* = 2 donors). Scale bars = 20 µm. (C, D) Viable PCs were quantified by flow cytometry and related to the number of PCs seeded at day 0, (C) and absolute amounts of secreted immunoglobulins for IgG1, IgA, and IgM were measured by multiplex immunoassay on indicated days (D). (E) Percentage of viable CD19^−^ and CD19^+^ CD38^+^CD138^+^ PCs on day 0 versus day 7 or 21 cultured in 2D measured by flow cytometry. Data in C‐E represent *n* = 5–10 donors as indicated by symbols. Horizontal lines indicate mean with SD. *p*‐values were calculated using the Mann–Whitney (C, D) or Wilcoxon matched‐pairs signed rank (E) test.

To provide the niche factors required for plasma cell survival, MSCs and PCs were either co‐cultured in classical 2D cell culture dishes or a 3D ceramic scaffold. To allow settling in the more complex 3D scaffold, MSCs were seeded onto the scaffold 7 to 10 days prior to PC seeding (Figure 2A). Immunofluorescence of fibronectin (FN1) and actin staining using phalloidin showed the typical cellular structures associated with stromal cells during the MSC/PC co‐culture, indicating the continuous presence of MSCs in the scaffold (Figure 2B).

To assess the capacity of MSCs to support PC survival in vitro, CD38^+^CD138^+^ PCs were isolated and cultured with or without MSCs for 21 days. After 7, 14, or 21 days, DAPI^−^CD38^+^CD138^+^ PCs were identified by flow cytometry (Figure 2C). While the percentage of viable PCs cultured alone without MSC contact continuously decreased over the culture time, reaching 0.5% of the number of initially seeded PCs, the number of PCs were stably maintained over 21 days, when cultured with MSC contact in 2D and 3D (Figure 2C). A comparison of 2D and 3D conditions showed that there were no significant differences in survival.

To investigate whether the PCs surviving in the in vitro model remain functional and continuously secrete immunoglobulins, the total amount of IgG1, IgA, and IgM secreted by the cultured PCs on days 2, 7, 14, and 21 was determined by multiplex immunoassay. While on day 2, the immunoglobulin amounts were similar across the 3 conditions, the amount of all 3 immunoglobulins (Ig), IgG1, IgA, and IgM, decreased in the medium supernatant of PCs cultured in the absence of MSCs over time (Figure 2D), likely due to degradation or dilution by the medium exchange. Contrary, the amount of IgG1, IgA, and IgM secreted by the PCs cultured with MSC contact increased within the first week of culture, from day 2 to 7, and then remained relatively stable until day 21 (Figure 2D), despite repeated medium change, indicating continued immunoglobulin production. The amount of secreted immunoglobulins was comparable between the 2D monolayer and the 3D ceramic scaffold. Comparison of survival and antibody titers between 2D and 3D also suggested that there is likely a limitation in retrieving PCs from 3D scaffolds at the end of the experiment. The analysis of the population of viable CD19^−^, CD38^+^, and CD138^+^ PCs in the 2D co‐cultures indicated, that the percentage of CD19^−^ cells increased, while the percentage of CD19^+^ decreased (Figure 2E).

Taken together, data showed that human BM‐derived PCs required stromal cells to survive and sustain immunoglobulin secretion in vitro and that they can be long‐term maintained in 2D as well as the 3D BM model.

### 2.3 Survival of Human PCs Depends on Direct Cell–Cell Contact With MSCs

**FIGURE 3:**
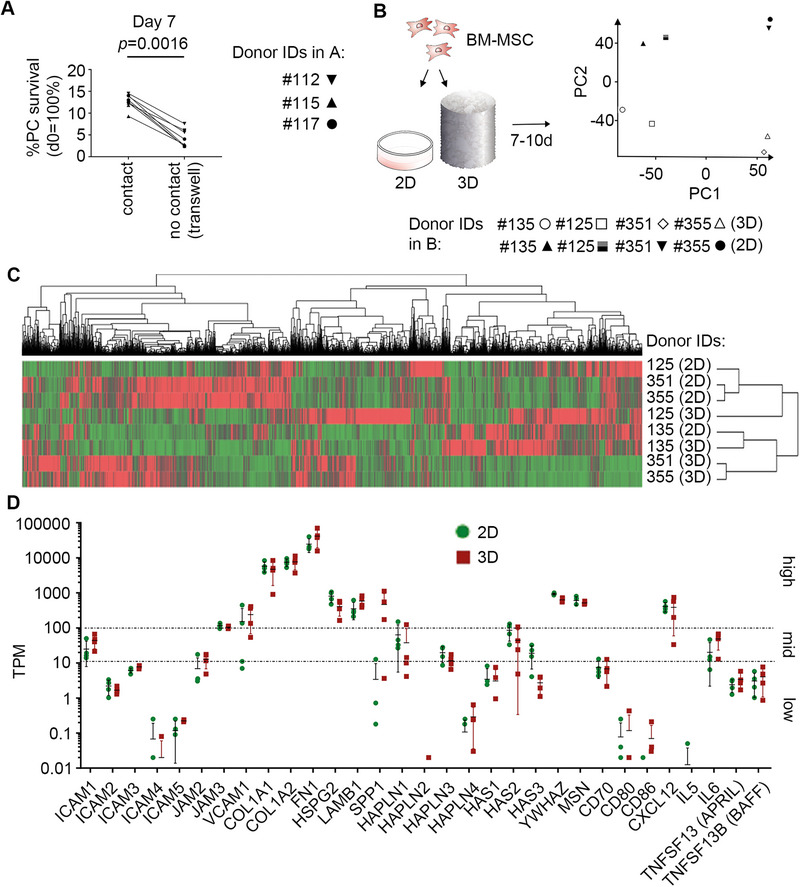
MSCs express relevant factors for the survival of BM LLPC populations, in both 2D and 3D culture conditions. (A) PC survival measured by flow cytometric analysis. Viable PCs were quantified by flow cytometry and related to the number of PCs seeded at day 0. PCs and MSCs were cultured either in direct cell–cell contact or separated by a transwell. Data in (A) represent *n* = 3 donors with technical replicates as indicated by symbols and lines correlate replicates. *p*‐values were calculated on the mean of the replicates for each donor using unpaired *t*‐test. (B) Scheme for preparing the 3D bone marrow ceramic scaffold pre‐culture with MSCs. Bulk RNA sequencing of MSCs of a confluent monolayer (2D), or MSCs pre‐cultured for 7 days in the ceramic scaffold (3D), *n* = 4 donors. (B, C) Principal component analysis and Heatmap cluster analysis of bulk RNA‐sequencing show clustering of the 2D and 3D cultures. (D) Transcripts per million (TPM) shown for selected genes previously indicated to be important in PC survival. The dotted lines indicate a classification of high, mid, and low expression of genes. Data from bulk RNA sequencing with *n* = 4 donors (mean with SD, statistical analysis was performed using Wilcoxon matched‐pairs signed rank test).

To investigate the mechanism of support by the MSCs, we first asked whether the human PCs, similar to murine PCs [13], required direct cell–cell contact with MSCs. Flow cytometric analysis of viable CD38^+^CD138^+^ PCs showed significantly reduced survival when PCs were cultured spatially separated from the MSCs in the transwell system compared to PCs cultured in direct contact with stromal cells of a monolayer (Figure 3A). This indicated that human PCs require direct cell–cell contact with stromal cells for their survival.

### 2.4 Sequencing Indicates PI3K and NF‐kappaB Signaling Pathways as Potential Regulators of PC Survival

To identify factors of MSCs relevant for PC survival, we performed bulk RNA sequencing of MSC confluent monolayers or MSCs pre‐cultured on the ceramic scaffold for seven days. Principal component analysis (Figure 3B) and cluster analysis (Figure 3C) showed prominent differences between the 2D and 3D cultures resulting in appropriate cluster formation. However, no significant differences in the expression level of known PC niche marker genes were detectable. MSCs expressed a range of such niche marker genes, including genes relevant for adhesion, chemotaxis, and signaling‐mediated PC survival (intercellular adhesion molecule (*ICAM1)*, vascular cell adhesion molecule (*VCAM1)*, *FN1*, osteopontin (*SPP1*), hyaluronan synthase (*HAS2)*, hyaluronan and proteoglycan link protein 1 (*HAPLN1)*, collagen 1 (*COL1*), laminin subunit beta 1 (*LAMB1*), *CXCL12*, tyrosine 3‐monooxygenase/tryptophan 5‐monooxygenase activation protein zeta (*YWHAZ)*, *IL‐6* (Figure 3D). As outlined above, many of these adhesion molecules and cytokines are known to signal via PI3K and NF‐kappaB. *APRIL* and *BAFF*, which have been described as key survival factors for PC survival in mice, were expressed by the stromal cells at low levels (Figure 3D). This indicated, that MSCs express PC niche‐relevant genes in both 2D and 3D conditions.

### 2.5 Survival and Function of Human PCs Depend on PI3K and Canonical NF‐kappaB Signaling

**FIGURE 4:**
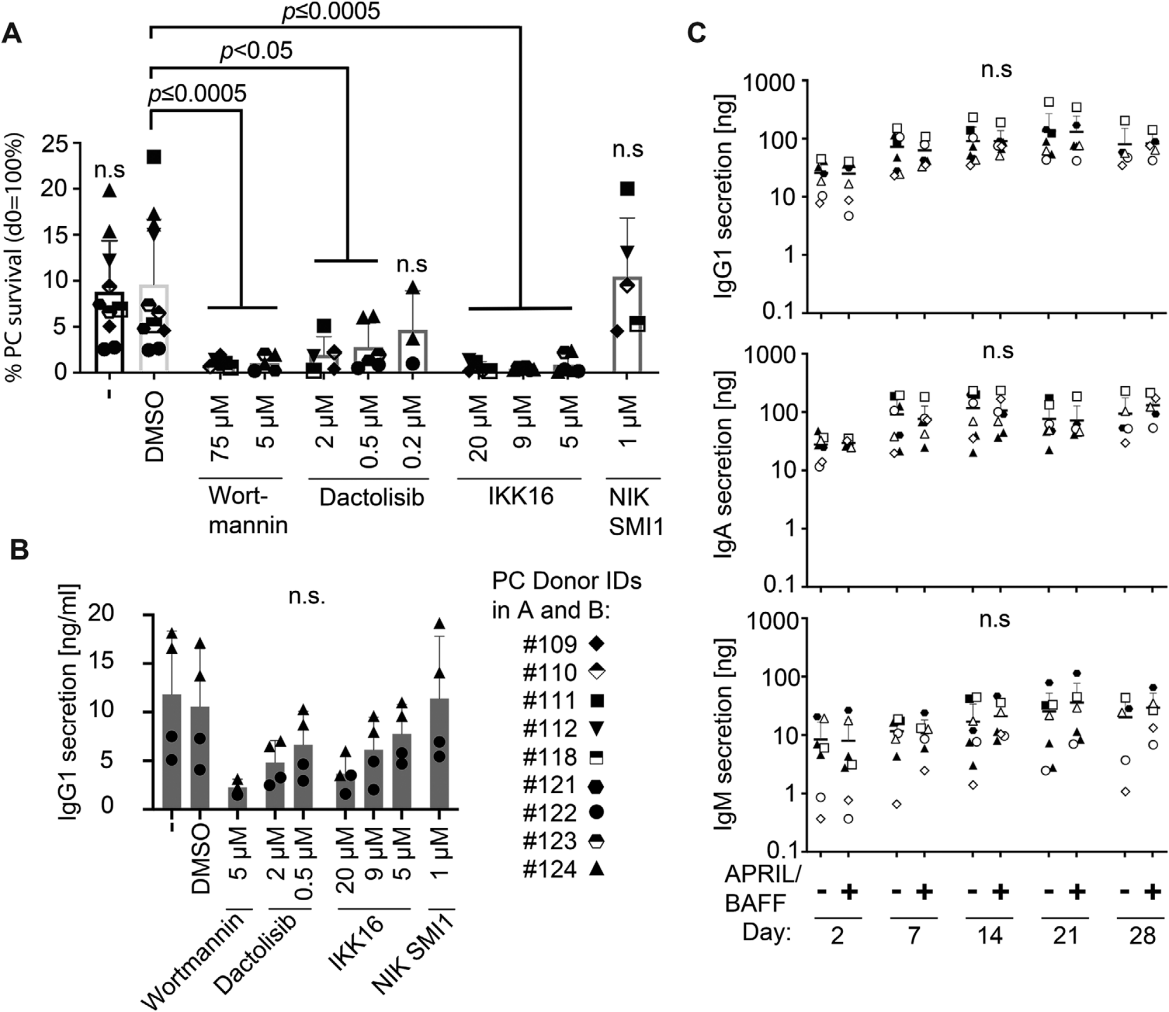
Human bone marrow plasma cell survival depends on PI3K and canonical NF‐kappaB signaling but not on exogenous APRIL and BAFF. (A) Quantification of viable CD38^+^CD138^+^ BM PCs by flow cytometry 2 days after treatment. PCs were cultured in 2D and treated on day 0 with indicated concentrations of the PI3K inhibitors Wortmannin and Dactolisib or the NF‐kappaB pathway inhibitors IKK16 or NIK SMI1. Vehicle control with 0.1% DMSO. Bars represent mean with SD of *n* = 3–9 donors as indicated by symbols. (B) Concentrations of IgG1 secreted by PCs cultured in 3D were measured by multiplex immunoassay on day 2 after treatment with indicated concentrations of inhibitors. Bars represent mean with SD of *n* = 3–4 with individual symbols representing 2 PC donors each combined with 1–2 MSC donors. (C) Quantification of absolute amounts of IgG1, IgA and IgM secreted by PCs cultured in 3D without or with exogenous APRIL (25 ng/mL) and BAFF (1.2 ng/mL) measured by multiplex immunoassay on indicated days. Horizontal lines represent mean with SD of *n* = 5–6 donors as indicated by symbols. *p*‐values were calculated using the Mann–Whitney test, comparing vehicle control (DMSO) with the treated samples in (A) and (B) and untreated with APRIL and BAFF for each time point in (C).

Since the RNA sequencing highlighted signaling through PI3K, and NF‐kappaB pathways, and these pathways previously were suggested to be crucial for the survival of mouse PCs [13], we tested the importance of these pathways for human PCs in our model. Flow cytometric analysis of CD38^+^CD138^+^ PCs of PC‐MSC co‐culture in a 2D monolayer revealed a significant reduction in survival on day two when treated with the PI3K inhibitor Wortmannin at both tested concentrations (5 and 75 µM; Figure 4A). Inhibition with the dual‐PI3K inhibitor Dactolisib showed a dose‐dependent effect on PC survival (Figure 4A). Inhibition with the NF‐kappaB inhibitor IKK16 also significantly reduced PC survival at the tested concentrations (5–20 µM; Figure 4A). However, inhibition with NIK SMI1, targeting the noncanonical NF‐kappaB pathway, showed no impact on PC survival (Figure 4A). IgG1 concentrations in the medium supernatant of cultured PCs measured with multiplex matched the survival (Figure 4B). Both assays, survival measured by flow cytometry (Figure 4A) and antibody secretion measured by multiplex immunoassay (Figure 4B), are not directly comparable because they are performed either on 2D or 3D cultures, respectively. However, since both assays give a similar result, this indicates that PI3K and canonical NF‐kappaB pathways are involved in functional PC survival. Inhibitors also impacted MSC survival and niche marker expression, opening the possibility, that the decrease of PC survival may at least in part be caused by indirect effects. However, Wortmannin, Dactolisib, and IKK16 at the lowest concentration (5 µM) showed no significant effect on stromal cell survival yet significantly affected PC survival (Figure S1). While niche marker expression of *CXCL12* and *VCAM1* was affected by IKK16 and Dactolisib, none of the niche marker expression was affected by Wortmannin, underlining a likely direct effect by this inhibitor on the PCs (Figure S2).

In murine PC in vitro culture, exogenous addition of APRIL and BAFF is necessary [13, 18, 19, 25] yet the RNA sequencing of MSCs had indicated that the human MSCs may express these crucial factors already (Figure 3D), pointing to the possibility, that in human culture, the external addition of these factors may not be necessary. Indeed, the addition of APRIL and BAFF to the culture medium did not change human PC survival in the 3D co‐culture, as indicated by Ig multiplex immunoassay (Figure 4C). Together, the data indicated dependence of functional PC survival on PI3K and canonical NF‐kappaB. Contrary to the murine system, human MSCs may either provide sufficient APRIL and BAFF so that the cultures are saturated, or human BM PCs may not depend on APRIL and BAFF.

### 2.6 The 3D BM Model Is Compatible With Microfluidic Multi‐Organ‐Chips

**FIGURE 5:**
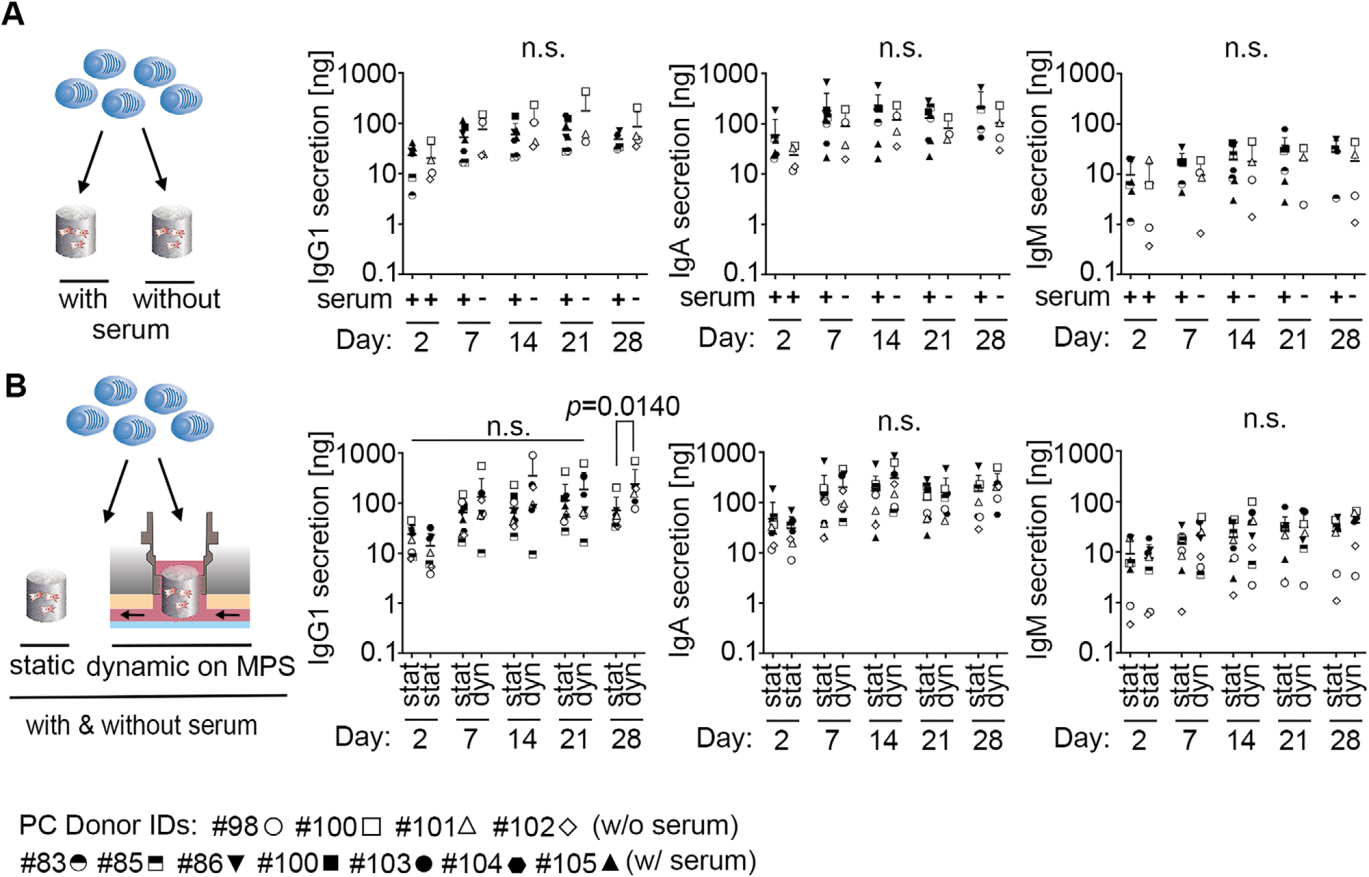
The 3D BM model enables the culture of the BM PC niche in a serum‐free medium and the integration into a microfluidic multi‐organ‐chip. (A) Quantification of absolute amounts of IgG1, IgA, and IgM secreted by PCs cultured in 3D with or without serum from day 2 onwards, measured by multiplex immunoassay on indicated days. (B) Quantification of absolute amounts of IgG1, IgA, and IgM secreted by PCs cultured in 3D under static (stat) or dynamic (dyn) conditions in a microphysiological system from day 2 onwards, measured by multiplex immunoassay on indicated days. (A, B) Horizontal lines represent mean with SD. Data represent *n* = 3–6 (A) and *n* = 6–8 (B) donors as indicated by symbols. *p*‐values were calculated using the Mann–Whitney test, comparing samples for each time point. In some cases, experiments in Figure 5A,B and Figure 4C were run in parallel and from these experiments, pooled data from Figure 5A are used as the static control in Figure 5B and as untreated control in Figure 4C.

The PC culture methods described here can be used to analyze the PC niche and effects of drugs. To enhance usability, we also tested the compatibility of the PC culture with our previously published long‐term culture of human hematopoietic stem and progenitor cells (HSPC) BM model [26] in a microphysiological system (MPS). The HSPC BM model necessitates a serum‐free culture medium to maintain HSPCs in a native state. Analysis of immunoglobulin secretion showed that PC survival and function are not dependent on serum over 28 days of culture (Figure 5A), indicating the compatibility of the two models. Analysis of immunoglobulins in either static or dynamic conditions in the multi‐organ‐chip showed comparable total amounts of secreted immunoglobulins IgG1, IgA, and IgM during 28 days of culture (Figure 5B). This indicated that PCs cultured in the 3D BM model could be maintained equally under static conditions in a 24‐well format and dynamic conditions in the MPS, highlighting the feasibility of culturing the 3D BM PC niche model within the MPS and offering versatile opportunities for future applications.

## 3 Discussion

The LLPC is a central part of the humoral memory and an important driver in autoimmune disease. While murine LLPCs can be studied in vivo and in vitro, human LLPCs are difficult to study because of very limited accessibility. Here, we present the isolation of LLPCs from the human femoral head and culture conditions to maintain LLPCs and their antibody‐secreting function for 21 days in vitro. Confirming the data obtained for murine LLPCs [5, 13, 21, 27], the culture of human LLPCs depends on contact with MSCs, PI3K, and NF‐kappaB signaling, but not exogenous addition of BAFF and APRIL.

The scarcity of human in vitro models for the BM PC niche can be attributed to several reasons. Plasma cells are a rare cell type, constituting, on average, 0.25% of nucleated bone marrow cells [7]. Protocols to isolate cells from human bone marrow exist [22, 28, 29, 30], but human BM extracts are very limited. Therefore, we and others isolated BM PCs from the femoral head of hip replacement surgeries [31]. Once isolated, plasma cells are fragile, highly dependent on their microenvironment, and prone to apoptosis due to endoplasmic reticulum and mitochondrial stress [13, 32]. For these reasons, studies on human LLPCs have been primarily conducted using blood‐derived, in vitro‐generated human plasma cells [22, 23, 24]. The here‐presented isolation and culture strategy provides means to obtain high numbers of human primary LLPCs, which will promote future analysis.

While the markers CD38 and CD138 are commonly used for BM PCs [33, 34] the importance of CD19 is still under debate, with data indicating that the absence of CD19 defines LLPCs [29, 35] and other data challenging this definition [36, 37]. Recent data from single‐cell RNA sequencing identified that CD19 expression was present in early‐stage BM PC subsets but absent in activated late‐stage LLPCs [28]. Further, CD19 was shown to be downregulated in response to IL‐21 in activated B cells. CD19^low^ PCs were suggested to be generated in IL‐21‐dependent follicular germinal center reactions, while CD19^high^ PCs would be generated in IL‐21 independent extrafollicular activations of B cells [31]. In our experiments, freshly isolated PCs showed a heterogeneous expression of CD19, as observed before [29, 35]. This could indicate the presence of PCs at different stages of maturity. Interestingly, after 21 days, we observed a significant shift toward CD19^−^CD38^+^CD138^+^ PCs. The observed decrease of CD19‐expressing PCs over the culture time could be explained by two possibilities: either a transition of CD19^+^ to CD19^−^ BM PCs, as observed by others [38], or this shift could indicate an enhanced intrinsic survival capability among the CD19^−^ PC subsets or a selection of CD19^−^ PC subsets through unknown microenvironmental cues that are present in our model.

The isolation process used here cannot exclude the presence of other supportive cells, such as myeloid cells, which could potentially contribute to PC survival [39, 40]. However, MSC RNA sequencing did not indicate the expression of respective marker genes (data not shown). The low survival rates in PC single cultures indicated that any possible other population present after isolation is insufficient for PC support.

Niche factors presented by MSCs have been described to include cell–cell interactions and soluble factors [6, 13, 22, 23, 24, 30]. While our data confirm the necessity of MSC cell contact for PC survival, this does not inform about the importance of secreted factors, since secreted factors may only function upon established cell–cell contact. Extracellular matrix (ECM) components such as FN1, hyaluronic acid (HA), COL, and SPP1, highly expressed in the MSCs used in this study, are known factors for PC homing and maintenance. FN1 acts as a network to capture and retain secreted factors and serves as a ligand for surface receptors expressed on PCs, including CD138 [24]. HA is the primary ligand for PC CD44 [41, 42]. The interaction of CD44 on PCs with the ECM components secreted by MSCs or direct contact with MSCs has been demonstrated to induce the production of IL‐6 by MSCs, which functions as a further PC survival factor [42]. Furthermore, the RNA sequencing data showed that genes (*CXCL12, VCAM1, ICAM1, YWHAZ, IL‐6, APRIL, BAFF*) known to play a crucial role in PC chemotaxis and homing [12, 43, 44], and long‐term survival in the bone marrow [5, 13, 18, 21, 23, 24, 45] were expressed by the MSCs at different levels. Inhibitor experiments confirmed the PI3K and NF‐kappaB pathways central for LLPC survival and function. From mouse models, it is known that integrin‐mediated cell contact between PCs and MSCs can activate the PI3K‐signaling pathway promoting PC survival in the BM [13, 46]. Moreover, the NF‐kappaB signaling pathway was shown to support plasma cell survival by its downstream induction of the antiapoptotic molecule MCL‐1 [13, 21, 47].

Taken together, we present isolation of human BM PC, in vitro culture for up to 28 days with enrichment of CD19^−^ cells over time. In our model, primary BM MSCs stably express a set of factors that have been previously described to be relevant for the BM niche, indicating that they created a supportive microenvironment for the long‐term culture of PCs. The 2D and 3D culture systems were equally effective in maintaining BM PCs. Yet, the 3D dynamic culture system on chip offers the possibility to culture the BM model in interaction with other models, for example, for applications including drug or substance testing in the future.

## 4 Methods

### 4.1 Human Samples

Human cells were isolated from the femoral heads of patients undergoing hip joint replacement surgeries at the Immanuel Krankenhaus in Berlin‐Wannsee and Jüdisches Krankenhaus in Berlin‐Wedding, Germany. This study was approved by the ethics committee of the Charité Universitätsmedzin Berlin, Germany, and informed consent was obtained from all donors (1‐8001602‐01‐EF and EA4/072/20).

### 4.2 Isolation of BM PCs

Human PCs were isolated on the same day of the surgery. Femoral heads were fragmented by mechanical preparation. Bone marrow pieces were rinsed thoroughly with phosphate‐buffered saline supplemented with 3% bovine serum albumin and 5 mM ethylenediaminetetraacetic acid. The collected cell suspension was filtered through a 100 µM strainer, and mononuclear cells (MNCs) were separated through density gradient centrifugation. PCs were separated from other MNCs by a two‐step magnetic sorting, first using the non‐plasma cell biotin‐antibody and microbead cocktail (without the CD56 microbeads) of the plasma cell isolation kit II (130093628, Miltenyi Biotec, Germany) to separate non‐PCs. PCs were subsequently enriched from the negative fraction of the first sorting step using CD138 microbeads (130051301, Miltenyi Biotec). Cell number and purity were assessed by flow cytometry according to viability and CD38 and CD138 expression. Freshly isolated PCs were centrifuged at 300 x g for 5 min before they were resuspended in a culture medium and directly added to the respective culture condition. PCs were either cultured in serum‐containing Roswell Park Memorial Institute 1640 (10040CV Corning, USA) + 10% fetal bovine serum (35079CV, Corning) + 1% Penicillin–Streptomycin (P/S; 30002CI, Corning) or in serum‐free StemSpan–animal origin‐free medium (1000130, Stemcell Technologies, Canada) + 25 ng/mL Fms‐related tyrosine kinase 3 ligand (30019, PeproTech, USA) + 10 ng/mL thrombopoietin (30018, PeproTech) + 1% P/S. APRIL and BAFF were added at concentrations of 25 ng/mL of APRIL (5860AP, R&D Systems, USA) and 1.2 ng/mL of BAFF (7537BF, R&D Systems) according to the manufacturer's recommendations.

## Author Contributions

Conceptualization: Mark Rosowski, Roland Lauster, Zehra Uyar‐Aydin and Sina Bartfeld. Investigation: Zehra Uyar‐Aydin. Formal analysis: Zehra Uyar‐Aydin and Mark Rosowski. Supervision: Mark Rosowski, Sina Bartfeld, and Shirin Kadler. Funding: Roland Lauster and Sina Bartfeld. Visualization: Zehra Uyar‐Aydin, Mark Rosowski, and Sina Bartfeld. Writing–original draft: Zehra Uyar‐Aydin, Sina Bartfeld, and Mark Rosowski. Writing–review and editing: All authors.

## Conflicts of Interest

The authors declare no conflicts of interest.

## Supporting information



Supporting Information

## Data Availability

The data that support the findings of this study are openly available in Gene Expression Omnibus (GEO) under accession number GSE277163.
