# Supplementary material for: Survival of Human Bone Marrow Plasma Cells In Vitro Depends on the Support of the Stromal Cells, PI3K, and Canonical NF‐kappaB Signaling
Source: Eur J Immunol. 2025 Jan 8;55(1):e202451358. doi: 10.1002/eji.202451358 (PMC11708448; doi:10.1002/eji.202451358)
Supplement: Supplementary file 1 — Supporting Information [file EJI-55-e202451358-s001.pdf]

Survival of human bone marrow plasma cells *in vitro* depends on the support of the stromal cells, PI3K and canonical NF-kappaB signaling.

Zehra Uyar-Aydin[1], Shirin Kadler[2], Roland Lauster[1,2], Sina Bartfeld[#1,2], Mark Rosowski[#1,2]

[1] Technische Universität Berlin, Institute of Biotechnology, Department Medical Biotechnology, Berlin, Germany
[2] Si-M/ Der Simulierte Mensch, Technische Universität Berlin and Charité Universitätsmedizin Berlin, Berlin, Germany
# authors contributed equally
Correspondence to: s.bartfeld@tu-berlin.de and mark.rosowski@tu-berlin.de

Supplementary Material

- **Supplementary Figures**
- **Supplementary Experimental Procedures**
- **Supplementary References**

SUPPLEMENTARY FIGURES

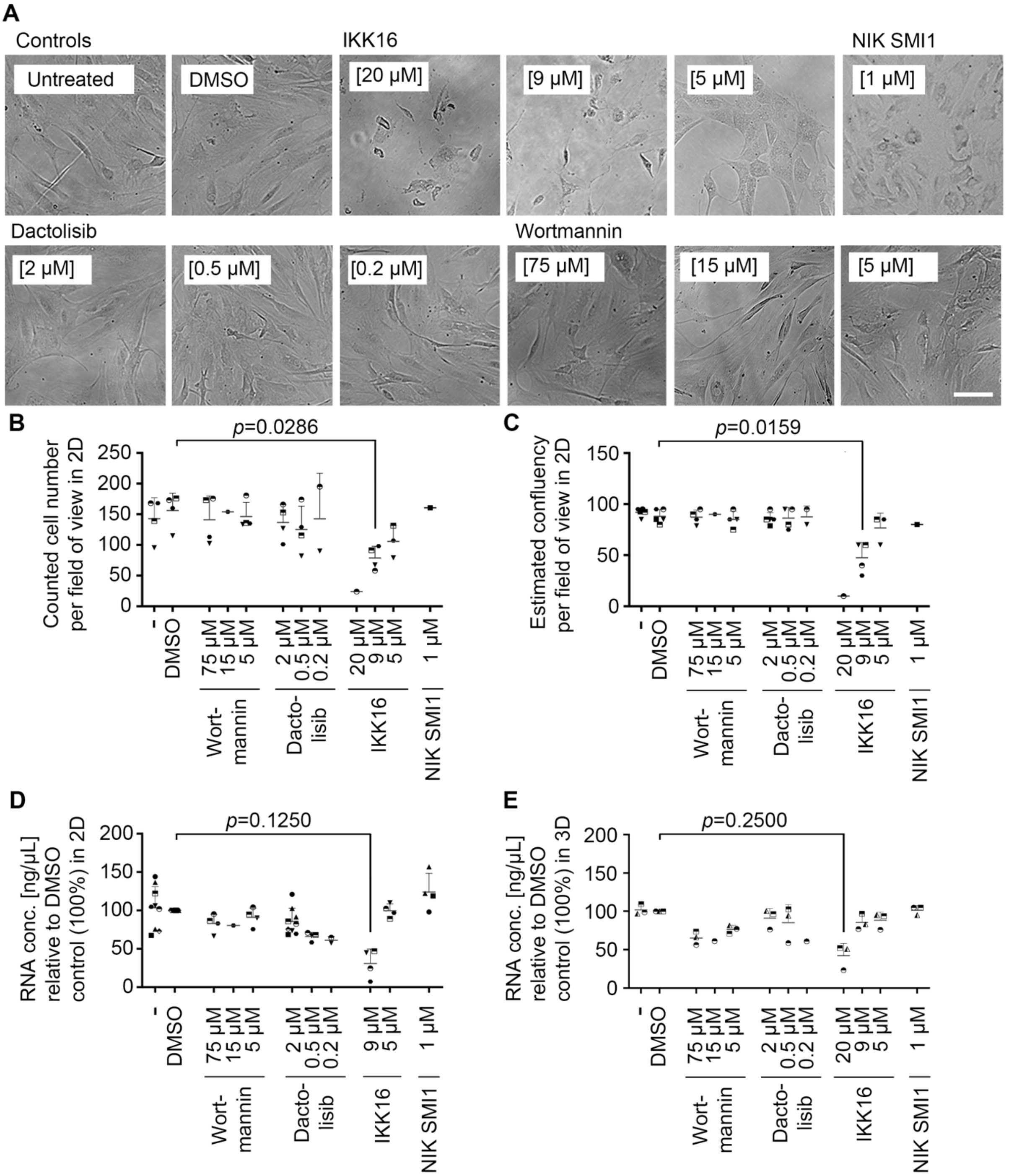

Figure S1: Addition of the inhibitor IKK16 decreased MSC survival at high concentrations, but IKK16 at lower concentrations and other inhibitors showed no significant effect on stromal cell morphology, cell count, monolayer confluency in 2D and RNA yield in 2D and 3D. MSCs were seeded and pre-cultured in 2D (**A–D**) or the 3D scaffold (**E**). Inhibitors were added at the indicated concentrations for two days before cells were imaged, counted and confluency was estimated (**A–C**), or cells were lysed, and RNA was isolated. The RNA concentrations were determined spectrophotometrically at 260 nm and normalized to the RNA concentration of the DMSO control for each donor (**D, E**). Horizontal lines represent the mean with SD of n = 1–8 donors as indicated by individual symbols (**B–E**). P-values were calculated compared to the vehicle control with DMSO using Mann-Whitney test (**B, C**) or Wilcoxon matched-pairs signed rank test (**D, E**). Scale bar in A: 100 µm.

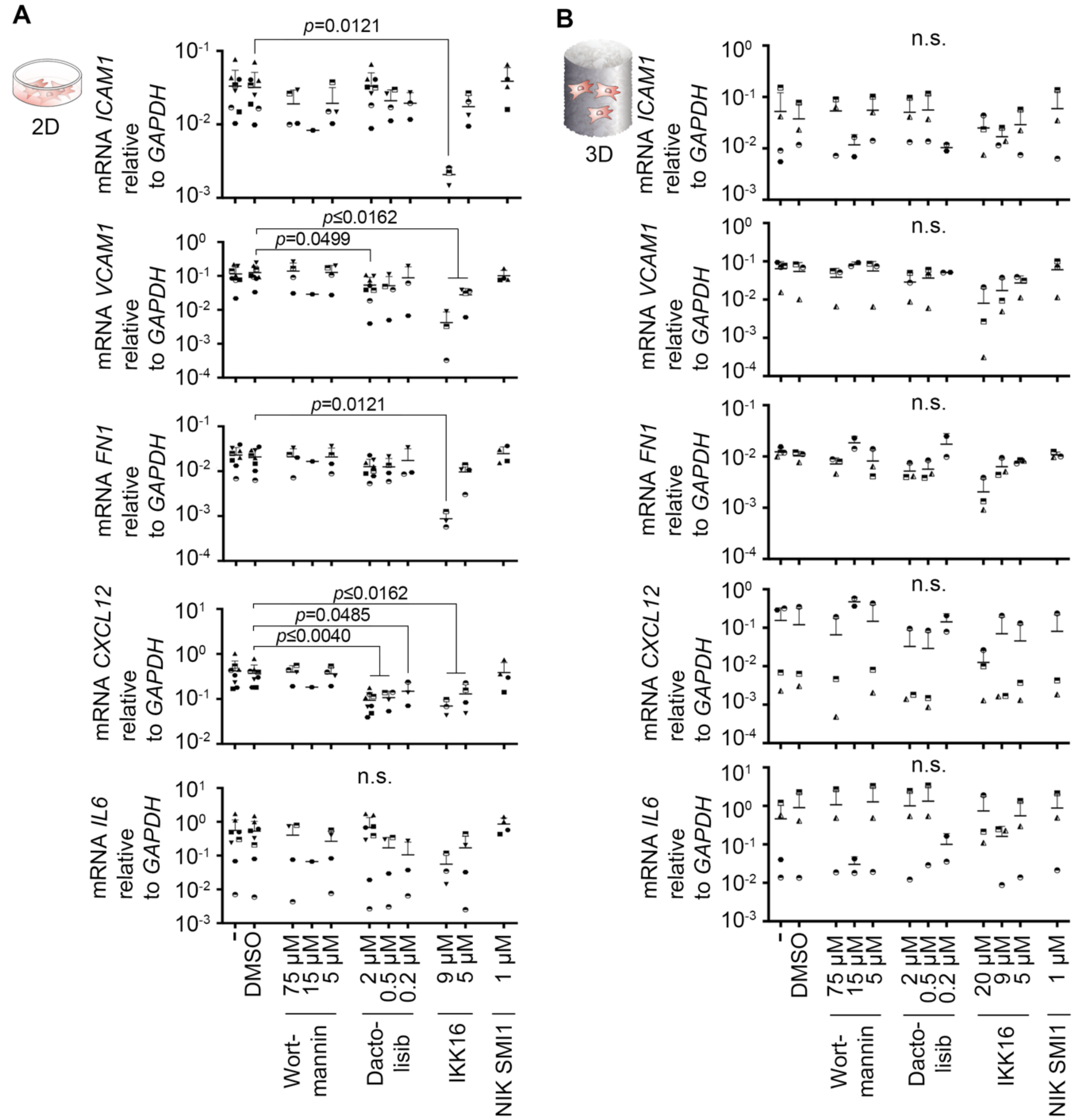

Figure S2: In 2D, stromal cell niche marker expression was significantly affected by Dactolisib and IKK16, but not by Wortmannin. In 3D, inhibitors showed no significant effect on stromal cell niche marker expression. MSCs were seeded, pre-cultured, and treated with inhibitors at the indicated concentrations for two days in 2D (**A**) or the 3D scaffold (**B**) before lysis, RNA isolation, and qRT-PCR analysis of the indicated genes. Horizontal lines represent the mean with SD of (**A**) n = 1–7 donors as indicated by individual symbols and (**B**) n = 2–4 donors as indicated by individual symbols. P-values were calculated compared to the vehicle control with DMSO using Mann-Whitney test.

SUPPLEMENTARY EXPERIMENTAL PROCEDURES

Isolation and expansion of MSCs

Human MSCs were isolated from the bone marrow of femoral heads, as described before (Sieber et al., 2018). In brief, the isolation of MSCs required a thorough initial rinsing of the primary tissue with phosphate-buffered saline (PBS), followed by density gradient centrifugation and expansion of the MSCs through their ability to adhere to plastic surfaces in Dulbecco's modified Eagle's medium (DMEM; 10013CV, Corning, USA) + 10% fetal bovine serum (FBS) + 1% Penicillin–Streptomycin (P/S). MSCs were used until passage 6.

2D and 3D culture systems

For the 2D and 3D PC-MSC co-culture systems, primary human MSCs and PCs of different biological donors were used. Each PC donor was paired with a different MSC donor to increase biological variability. While isolated human MSCs needed to be expanded prior to their use, isolated human plasma cells were used immediately upon isolation.

For 2D co-culture experiments, freshly isolated human plasma cells were co-cultured with MSC monolayers. MSCs were seeded into a 12-well plate at a density of 50 000 cells per well either on the same day or one to two days before the start of the experiment and cultured in DMEM + 10% FBS + 1% P/S of a volume of 2 mL. Immediately before the plasma cells were added the medium was changed to Roswell Park Memorial Institute (RPMI) 1640 + 10% FBS + 1% P/S for PC culture, and 5000 plasma cells were added. For 3D co-culture experiments, hydroxyapatite-coated zirconium oxide-based Sponceram 3D ceramics (Zellwerk GmbH, Germany) with 5.8 mm in height and diameter were used as scaffolds. These ceramics were first seeded with MSCs at a density of 500 000 cells per scaffold in an ultra-low attachment (ULA) 96-well plate (3474, Corning, USA). After 6 hours, the ceramics were transferred to a 24-well ULA plate (3473, Corning, USA) and cultured in DMEM with 10% FBS and 1% P/S for 7–10 days. Before PCs were added, the remaining DMEM + 10% FBS + 1% P/S culture medium from the inside of the pre-cultured ceramics was removed through quick spin-down, and 5000 plasma cells were carefully pipetted into the ceramic scaffold in several steps of small volumes. Afterwards, fresh RPMI 1640 + 10% FBS + 1% P/S culture medium in a total volume of 1200 µL was added immediately to completely immerse the ceramic scaffold in medium within the 24 well. Plasma cell monocultures in 2D were used as control conditions without stromal cell cultures, for which 5000 plasma cells were cultured in a 24-well ULA plate with 1 mL RPMI 1640 + 10% FBS + 1% P/S culture medium. For transwell experiments, transwell inserts of 0.4 µm pore size for 24 well plates (662641 and 662640, Greiner Bio-One, Austria) were used. The transwell polyester inserts were seeded with MSCs at a density of 30 000 cells per insert one or two days before the start of the experiment and cultured in DMEM + 10% FBS + 1% P/S. The culture medium was changed to RPMI 1640 + 10% FBS + 1% P/S medium before 5000 plasma cells were added to the bottom of the transwell plate in a volume of 850 µL. All cultures were kept at 37°C and 5% CO_2 with half-medium exchanges three times a week.

Treatment of plasma cells and stromal cells with inhibitors

Isolated PCs were treated on day 0 with inhibitors at different concentrations in RPMI 1640 + 10% FBS + 1% P/S. PCs were pre-treated with the irreversible PI3K inhibitor Wortmannin (S2758, Selleckchem, USA) at different concentrations for 20 minutes at 37°C before the inhibitor was washed out, and PCs were cultured for two days. Treatment with the PI3K inhibitor Dactolisib (S1009, Selleckchem, USA) and the NFKB inhibitors IKK16 (S2882, Selleckchem, USA) and NIK SMI1 (HY112433, MedChemExpress, USA) was followed by a 2-day culture in the presence of the inhibitors. As controls, cultures of untreated PCs and vehicle control PC cultures treated with the same concentration of DMSO (0.1%) were included.

To assess the effect of the inhibitors on stromal cells, stromal cell-only cultures in 2D or within the 3D scaffold were treated with inhibitors at different concentrations in RPMI 1640 + 10% FBS + 1% P/S in the same way as the PCs for two days. On day 2 after treatment, stromal cells cultured in 2D were imaged and lysed, and stromal cells cultured in 3D were directly lysed for qRT-PCR analysis.

Flow Cytometry

Isolated and cultured PCs were analyzed at different time points by flow cytometry. Cells were incubated with FCR blocking reagent (130059901) to block unspecific binding and stained with DAPI (130111570), CD38-APC (REA671), CD138-PE (44F9), and CD19-FITC (REA675) (all Miltenyi, Germany) for 10 minutes at 4°C at the manufacturer's recommended dilutions. Samples were washed with PBS supplemented with 3% bovine serum albumin and 5 mM ethylenediaminetetraacetic acid and analyzed using a MACSQuant Analyzer (Miltenyi, Germany) and either FlowJo v10 (Tree Star Inc., USA) or FlowLogic v8.7 (Inivai Technologies, Australia) software. $DAPI^-$ viable PCs were quantified based on their CD38 and CD138 expressions. For intracellular Ki67 staining, fixation and permeabilization were performed with eBioscience FoxP3 fixation and PermBuffer (00552300, Invitrogen, USA) after surface marker staining and according to the manufacturer's protocol. Cells were stained with Ki67-APC (REA183, Miltenyi, Germany, 1:50) for 30 minutes at room temperature in the dark.

Multiplex immunoassay

Multiplex immunoassay was used to determine secreted immunoglobulins (IgG1, IgA, IgM) in the supernatant of cultured PCs at different time points using the Bio-Plex Pro Human Isotyping Panel (171A3100M, Bio-Rad, USA) according to the manufacturer's instructions. Samples were analyzed using the Bio-Plex 200 system (Bio-Rad, USA). Secreted immunoglobulins are presented either as concentrations or as absolute amounts, calculated based on the total volume of the culture medium.

Immunofluorescence and confocal microscopy

Ceramic scaffolds pre-seeded with MSCs and cultured with PCs were fixed with 4% paraformaldehyde (PFA) for 30 min at 4°C. The scaffolds were rinsed in PBS three times for 5 minutes and cut in half using a precision saw (IsoMet 1000; Buehler, USA). Cut pieces of the

scaffold were transferred into a flat-bottom 96-well plate for staining. First, the scaffolds were incubated with 5% goat serum and 0.1% Trition-X-100 in PBS for 1 hour. Next, samples were partly stained with the primary antibody mouse anti-human fibronectin (MA511981, Thermo Fisher Scientific, USA) 1:100 diluted in 1% goat serum, 0.1% Triton-X-100, and PBS overnight at 4°C. Samples were then washed three times for 10 minutes and stained with the goat anti-mouse secondary antibody conjugated to CF488a (20010, Biotium, USA) 1:400 diluted in 1% goat serum, 0.1% Triton X-100, and PBS for 1 hour at room temperature (RT). Actin-stained samples were incubated with phalloidin-Atto 550 (19083, Sigma-Aldrich, Germany) 1:400 diluted in 1% goat serum, 0.1% Triton X-100, and PBS for 1 hour at RT. All incubation and washing steps were performed under agitation. The samples were washed in PBS three times for 10 minutes under agitation and analyzed using a confocal laser scanning microscope (Leica TCS SP8, Germany) equipped with HC PL APO CS2 63x/1.20 water objective and 488-nm and 552-nm diodes. Images were processed using ImageJ (National Institute of Health, NIH).

Cell count and confluency assessment in monolayer cultures

Brightfield images of stromal cell monolayer cultures treated with inhibitors at different concentrations, as well as untreated and vehicle controls, were used to assess the effect of inhibitors on stromal cell morphology and survival. Manual cell counting was performed on brightfield images at 10x magnification using the ‘Cell Counter’ plugin in Fiji (ImageJ2 v2.14.0). Confluency of the stromal cell monolayers was estimated from brightfield images at lower magnifications (2x or 4x) using the blind analysis tool in Fiji (ImageJ2 v2.14.0).

Microfluidic multi-organ-chip system

In this study, the multi-organ-chip (MOC) system HUMIMIC Chip 2 (TissUse, Germany), consisting of two independent circular channel systems, was used. Each circular channel system contains two culture compartments with a diameter equivalent to a 96-well which are connected through microfluidic channels, and a peristaltic on-chip micropump operated by air pressure to provide a pulsatile medium flow. The MOCs were fabricated as previously described by Wagner et al., (2013). In brief, a polycarbonate adapter plate was coated with a silicone rubber primer (Wacker primer G 790; Wacker Chemie, Germany) and incubated at 80°C for 20 minutes. For polydimethylsiloxane (PDMS; Sylgard 184 silicone elastomer Kit, 634165S, VWR, USA) replica molding, the adapter plate was then attached to a master mold within a casting frame, and PDMS was injected into this casting chamber followed by incubation at 80°C for 60 minutes. PDMS-free culture compartments and 500 µm thick PDMS pump membranes were formed using screws. Subsequently, the formed 2 mm thick PDMS layer, imprinted with channels of 100 µm height and 500 µm width, was permanently bonded to a 75 × 25 mm glass slide (Menzel, Germany) using low-pressure plasma oxidation (Femto, Diener Electronic, Germany) to form a fluid-tight microfluidic channel system.

The 3D ceramic scaffold was transferred to the culture compartment opposite the micropump of each circuit on day 2 after adding plasma cells, while the other compartment served as a medium reservoir. Both compartments of each circuit were filled with 400 µL

medium, and the medium flow was directed away from the scaffold, passing first through the medium reservoir.

RNA isolation and qRT-PCR

Cultured stromal cells in 2D and within the 3D ceramic scaffold treated with inhibitors at different concentrations, as well as their untreated and vehicle controls treated with 0.1% DMSO, were lysed on day 2 after treatment in the lysis buffer supplied by the manufacturer of the NucleoSpin RNA II kit (740955, Macherey-Nagel, Germany) supplemented with ß-mercaptoethanol. Total RNA was extracted using the NucleoSpin RNA II Kit according to the manufacturer's instructions. RNA concentrations were determined spectrophotometrically at 260 nm using a Nanodrop2000 (Thermo Fisher Scientific, USA). Reverse transcription of 100 ng total RNA was performed using the TaqMan Reverse Transcription Reagents (N8080234, Applied Biosystems, USA) according to the manufacturer's protocol. Real-time PCR for quantitative analysis of cDNA expression was performed using the SensiFast SYBR No-ROX Kit (BIO98020 Bioline, UK) with 1 µL cDNA and 0.25 µM of each primer in 96-well PCR plates (710876, Biozym Scientific, Germany), on a CFX Opus 96 Real-Time PCR System (Bio-Rad, USA). Melting curve analysis was performed after each run to confirm specificity. Gene expression levels were calculated using an amplification efficiency (E) of 1.95 and normalized to the reference gene glyceraldehyde 3-phosphate dehydrogenase (GAPDH). Primers used in this study were: *ICAM1* forward 5'-CCGACTGGACGAGAGGGATT-3' and reverse 5'-TCGGCCCGACAGAGGTAGGT-3', *VCAM1* forward 5'-ACCCAAACAAAGGCAGAGTACG-3' and reverse 5'-CCACAGGATTTTCGGAGCAG-3', *FN1* forward 5'-CAGACCTATCCAAGCTCAAGTGG-3' and reverse 5'-TGGGTGGGATACTCACAGGTC-3', *CXCL12* forward 5'-GAGCTACAGATGCCCATGCC-3' and reverse 5'-AGCTTCGGGTCAATGCACAC-3', *IL-6* forward 5'-AAGCAGCAAAGAGGCACTGG-3' and reverse 5'-TGGGTCAGGGGTGGTTATTG-3', *GAPDH* forward 5'-TGTTGCCATCAATGACCCCTT-3' and reverse 5'-CTCCACGACGTACTCAGCG-3'.

Next-generation sequencing for transcriptome analysis

Transcriptome analysis of cultured BM stromal cells in 2D and 3D was performed using next-generation sequencing (NGS) on the MiSeq and NovaSeq X Plus platforms (Illumina, USA).

The cDNA library for samples from two donors, for analysis on the MiSeq platform, was prepared using the TruSeq Stranded mRNA LT Sample Prep Kit (RS1222101, Illumina, USA), following the TruSeq Stranded mRNA Sample Preparation Protocol LS (Illumina, USA). The initial quantity of total RNA was 800 ng for both stromal cells cultured in monolayers and 3D scaffolds. Multiple purification steps were performed using Mag-Bind RxnPure Plus magnetic beads (M1386, Omega Bio-tek, USA) to separate nucleic acids from the reaction mixtures during library preparation. For quality control, the concentration and fragment size of the cDNA were determined using a NanoDrop2000 spectrophotometer and gel electrophoresis (2% agarose). PCR was performed to verify the purity of the mRNA-based library and detect any contamination with genomic DNA or ribosomal RNA. The sequencing was performed on the Illumina MiSeq system.

For analysis on the NovaSeq X Plus platform, total RNA samples from an additional two BM stromal cell donors were quantified using a Qubit Fluorometer, and RNA integrity was checked on a TapeStation (Agilent Technologies, USA). Double-indexed stranded mRNA-Seq libraries were prepared using the ILMN Stranded mRNA Library Prep Kit (20040534, Illumina, USA), starting from 500 ng of input material for both stromal cells cultured in monolayers and 3D scaffolds, according to the manufacturer's instructions. Libraries were equimolarly pooled based on Qubit concentration measurements and TapeStation size distributions. The loading concentration of the pool was determined using a qPCR assay (7960573001, Roche, Switzerland). Libraries were then sequenced on the Illumina NovaSeq X Plus platform using PE100 sequencing mode, with a target of 50 million reads per library.

Raw data generated by the Illumina MiSeq and NovaSeq X platforms were processed using the Galaxy Project Platform (The Galaxy Community, 2024). The output data were converted to Sanger-compatible format using the FASTQ Groomer tool. The sequencing reads were mapped to the human genome reference (hg38) using HISAT2 to detect splice junctions between exons. Mapped reads were then quantified using the featureCounts tool on the Galaxy Project platform. Differential expression analysis was conducted using the DESeq2 algorithm (nominal p-value < 0.05; fold change > 2) through DE analysis (https://yanli.shinyapps.io/DEApp/), provided by the Bioinformatics Core, Center for Research Informatics (CRI), Biological Sciences Division (BSD), University of Chicago. Principal component analysis (PCA) and heatmap plots were generated using the ClustVis tool (Metsalu & Vilo, 2015) and the Morpheus platform from Broad Institute (https://software.broadinstitute.org/morpheus). RNA sequencing datasets generated in this study are available in the Gene Expression Omnibus (GEO) under accession number GSE277163.

Statistical testing

Data are presented as mean with standard deviation (SD). Generally, each donor is represented by one symbol. If a symbol is used twice in the same figure regarding MSCs, we used the MSCs from a donor in independent experiments, e.g. in a different week. Unless otherwise stated, if a symbol is used twice in the same figure regarding PCs, we combined PCs from the same donor with MSCs from different donors and thus considered the experiments as biologically independent. Data were tested for normality. For non-normally distributed data, Mann-Whitney or Wilcoxon matched-pairs signed rank test was performed to compare two groups, based on unpaired or paired data; for normally distributed data, according to the Shapiro-Wilk test, unpaired t-test was applied to compare two groups. Differences were considered statistically significant when p-values were ≤ 0.05. For further details, refer to the respective figure legend. All analyses were performed using GraphPad Prism v9.1.1 (GraphPad Software Inc., USA).

SUPPLEMENTARY REFERENCES

Metsalu, T., & Vilo, J. (2015). ClustVis: a web tool for visualizing clustering of multivariate data using Principal Component Analysis and heatmap. Nucleic Acids Research, 43(W1), W566–W570. https://doi.org/10.1093/nar/gkv468

Sieber, S., Wirth, L., Cavak, N., Koenigsmark, M., Marx, U., Lauster, R., & Rosowski, M. (2018). Bone marrow-on-a-chip: Long-term culture of human haematopoietic stem cells in a three-dimensional microfluidic environment. Journal of Tissue Engineering and Regenerative Medicine, 12(2), 479–489. https://doi.org/10.1002/term.2507

The Galaxy Community. (2024). The Galaxy platform for accessible, reproducible, and collaborative data analyses: 2024 update. Nucleic Acids Research, 52(W1), W83–W94. https://doi.org/10.1093/nar/gkae410

Wagner, I., Materne, E.-M., Brincker, S., Süssbier, U., Frädrich, C., Busek, M., Sonntag, F., Sakharov, D. a, Trushkin, E. V, Tonevitsky, A. G., Lauster, R., & Marx, U. (2013). A dynamic multi-organ-chip for long-term cultivation and substance testing proven by 3D human liver and skin tissue co-culture. Lab on a Chip, 13(18), 3538–3547. https://doi.org/10.1039/c3lc50234a
